# Does wing use and disuse cause behavioural and musculoskeletal changes in domestic fowl (*Gallus gallus domesticus*)?

**DOI:** 10.1098/rsos.220809

**Published:** 2023-01-25

**Authors:** Renée C. Garant, Bret W. Tobalske, Neila Ben Sassi, Nienke van Staaveren, Dan Tulpan, Tina Widowski, Donald R. Powers, Alexandra Harlander-Matauschek

**Affiliations:** ^1^ Department of Animal Biosciences, University of Guelph, 50 Stone Road E, Guelph, ON, Canada N1G 2W1; ^2^ Division of Biological Sciences, University of Montana, 32 Campus Drive, Missoula, MT 59812, USA; ^3^ Department of Biology, George Fox University, 414 N Meridian Street, Newberg, OR 97132, USA

**Keywords:** limb immobilization, flapping flight, muscle adaptations, resource use, keel bone damage

## Abstract

Domestic chickens may live in environments which restrict wing muscle usage. Notably, reduced wing activity and accompanying muscle weakness are hypothesized risk factors for keel bone fractures and deviations. We used radio-frequency identification (RFID) to measure duration spent at elevated resources (feeders, nest-boxes), ultrasonography to measure muscle thickness (breast and lower leg) changes, radiography and palpation to determine fractures and deviations, respectively, following no, partial (one-sided wing sling) and full (cage) immobilization in white- and brown-feathered birds. We hypothesized partially immobilized hens would reduce elevated resource usage and that both immobilization groups would show decreased pectoralis thickness (disuse) and increased prevalence of fractures and deviations. Elevated nest-box usage was 42% lower following five weeks of partial immobilization for brown-feathered hens but no change in resource usage in white-feathered birds was observed. Fully immobilized, white-feathered hens showed a 17% reduction in pectoralis thickness, while the brown-feathered counterparts showed no change. Lastly, fractures and deviations were not affected in either strain or form of wing immobilization; however, overall low numbers of birds presented with these issues. Altogether, this study shows a profound difference between white- and brown-feathered hens in response to wing immobilization and associated muscle physiology.

## 1. Introduction

Due to people's sedentary lifestyle and lack of bipedal locomotion in high-income countries [1], the relationship between physical activity and disease has come into the spotlight [2–4]. Physical activity affects all organ systems, such as the cardiovascular, respiratory and musculoskeletal systems [5]. To illustrate, the mechanical stimulus of muscle contractions on the periosteum increases blood flow and, as a result, stimulates the physical adaptation of muscles and bones (strength, shape, mass) in response to applied forces [6,7]. Thus, exercise to maintain strong bones and muscles is vital to protect the musculoskeletal system from injury [8,9]. A sedentary lifestyle, prolonged bed rest or limb immobilization can result in skeletal muscle and bone mass loss due to disuse and increase the risk of future injury [10–15].

By contrast to bipedal locomotion in humans, birds have two locomotor systems [16]. The forelimbs (wings) are used mainly for aerial locomotion (or underwater diving in species of penguins). The hindlimbs (legs) are used primarily for terrestrial locomotion, such as walking, jumping or swimming and are essential for controlled take-offs and landings [17,18]. The major forelimb muscles include the flight muscles: the pectoralis (downstroke and forward) and the supracoracoideus (upstroke), both of which attach onto the keel bone [16,19]. During flapping flight, the flight muscles will contract and shorten and produced forces transfer onto the keel bone [20].

The high-resistance exercise of skeletal muscles leads to muscle hypertrophy, while a lack of exercise leads to skeletal muscle atrophy [21]. This is particularly evident in species of waterfowl undergoing moult, where the simultaneous loss of all flight feathers renders birds flightless [22–25]. These birds will transition to terrestrial locomotion for approximately one month, show a reduction in bodyweight [26], and experience atrophy of the flight muscles and hypertrophy of the hindlimbs [22,27]. In a captive environment, birds may experience locomotor restrictions due to small enclosure sizes, feather clipping/pinioning or bandaging of a wing to heal a fracture or to protect from self-trauma. In these scenarios, a lack of mechanical load/resistance exercise on the wings could result in flight muscle atrophy. Muscle atrophy has an impact on bone metabolism and can contribute to bone loss [28], increase the risk of low-trauma fracture [29] and of falls [30]. To illustrate, a previously recommended form of flight restriction in parrots kept in captivity, one-sided wing feather clipping [31], can lead to impaired landing [32] and could theoretically lead to muscle injuries or bone fractures.

Galliformes, such as chickens kept for egg production (laying hens), are primarily terrestrial and use short burst flapping flight when disturbed or to roost [33] but also use their wings when performing comfort behaviours such as wing flapping [34]. These birds are prone to keel bone fractures and deviations, particularly from 25 to 35 weeks of age [35] as the growth and ossification of the keel bone continues [36,37]. The attached force-producing flight muscles probably play a role in fracture formation; however, the nature of this relationship is not currently understood. Keel bone fractures were originally believed to result from external factors, such as collisions within housing systems; however, this does not sufficiently explain the high occurrence of fractures in systems where birds have limited chances to collide [35]. Thøfner *et al.* [38] found a majority of keel fractures originated from within laying hens, and activity of the wings was suggested as a potential source of internal force [35]. Research on the mechanical loading of bones in Galliformes has focused primarily on bone properties of the humerus and tibia, with little focus on the keel or muscles from which the mechanical strain originates. Further, the degree to which mechanical loading is placed onto the keel depends upon the action of the wings. The housing system laying hens are kept in will greatly influence how the wings can be used [39]. Overcrowding and small housing units can restrict the ability of hens to flap their wings and aerially locomote. Escape and panic reactions may also result in vigorous and unequal wing flapping bouts, and associated power outputs could produce keel fractures [35]. Hypothetically, the asymmetric use of the wings could unequally distribute force and velocities onto the keel leading to a deviation or fracture in the direction of muscle action. Additionally, strain-specific differences are known to occur, with typically brown-feathered laying hens being heavier and less aerial compared with white-feathered laying hens [40–45], which needs to be considered.

To improve our understanding of wing use on the flight muscles and keel bone of two strains of laying hens, we immobilized one wing in a sling (partial immobilization) or moved hens to cages (full immobilization) for five weeks. We hypothesized that partial immobilization would reduce the usage of elevated resources, and thus reduce the thickness of the flight muscles (pectoralis and supracoracoideus) but increase the thickness of the lower leg muscles (*M. gastrocnemius pars medialis*, *M. fibularis lateralis* and *M. tibialis cranialis caput tibiale and femorale*) due to increased ground locomotion compared with non-immobilized birds. Furthermore, we hypothesized it would increase the presence and severity of keel bone fractures due to clumsy and uncontrolled take-offs and landings and increase the presence of keel bone deviations due to unequal wing use. Finally, we hypothesized that placing birds in cages where wing flapping and ground locomotion are severely impaired would produce more pronounced thickness decreases to both the flight muscles and lower legs, resulting in the increased presence of fractures due to a weakening of the bone associated with muscle disuse and produce no change in the presence of deviations, as hens would be unable to produce sufficient force due to wing immobilization.

## 2. Material and methods

### 2.1. Animals and housing

Sixty female domestic chickens (18 weeks of age) of two strains (30 white-feathered, 30 brown-feathered) were divided into 12-floor pens (5 hens/pen in total; strains mixed). Birds were housed as described by Garant *et al.* [45]. In brief, each pen (length × width × height: 183 × 244 × 290 cm) was bedded with wood shavings and contained two identical slatted platforms (length × width: 122 × 31 cm at a height of 70 cm) along adjacent pen walls, one perch attached to the long side of a platform (length 122 cm at a height of 70 cm), and a second perch spanning the width of the pen (length 244 cm at a height of 150 cm). Hens had ad libitum access to water from 12 nipple drinkers and feed in two 5 kg hanging feeders (Frandsen Corporation, North Branch, MN, USA). Similarly, within each pen, there were two individual nest-boxes. One of the feeders and nest-boxes were located on the ground while the second feeder and nest-box were placed on a platform (feeder on one, nest-box on the other platform). White, opaque boards were placed along adjoining pen walls to prevent physical contact and social learning between pens. Pens were within a ventilated, windowless room (14 : 10 L : D cycle) at the Research Station of the University of Guelph (Guelph, ON, Canada). The identity of each hen was marked with a plastic numbered leg band (20 mm), and a matching numbered silicone ‘backpack’ attached with two elastic straps and eyelets as described in Harlander Matauschek *et al.* [46].

### 2.2. Partial and full immobilization treatments

When hens were 25 weeks old, an equal number of brown- and white-feathered hens were randomly assigned to one of three treatments (10 white- and 10 brown-feathered hens/treatment): no immobilization (control, *n* = 20); partial immobilization (one wing slinged to restrict one locomotion system; 10 left wing, 10 right wing; *n* = 20) and full immobilization (housing in single cage to restrict two locomotion systems; wings and legs; *n* = 20). Non- and partially immobilized hens were mixed over eight pens (*n* = 5 per pen; strains and treatments mixed), while fully immobilized hens were transferred from four pens to individual cages.

**Figure 1.**
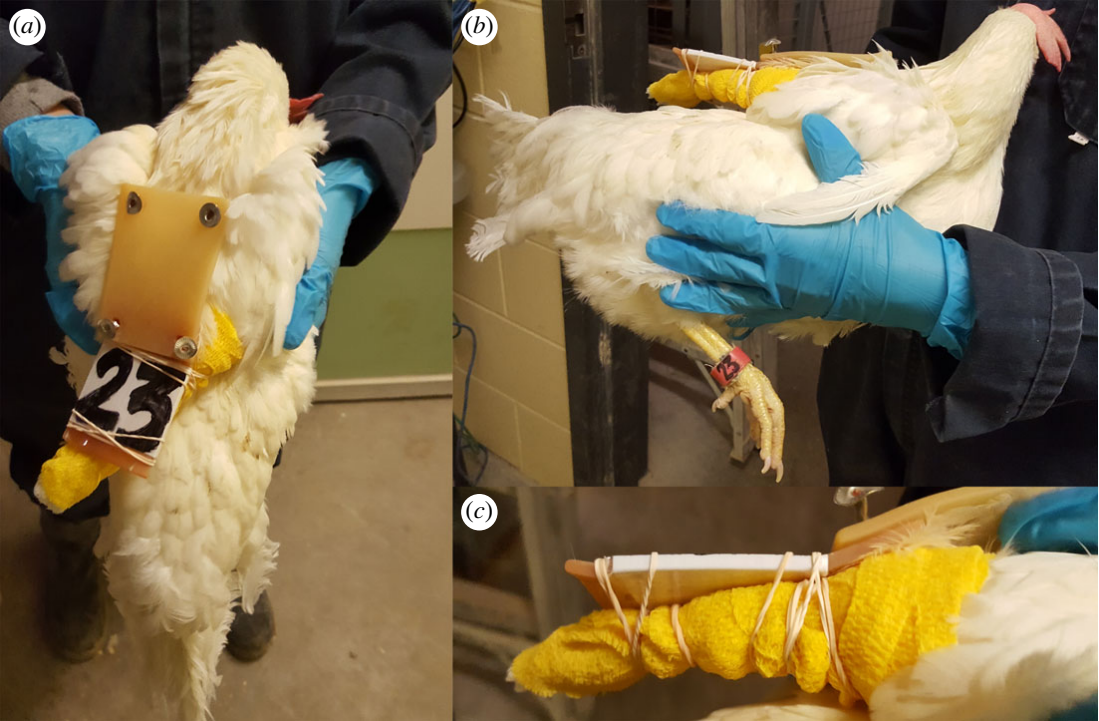
Series of three images displaying a hen with one wing slinged (partial immobilization). (*a*) Dorsal view of a right-wing immobilization via sling, (*b*) lateral view of a right-wing immobilization via sling and (*c*) close-up of one wing sling secured through 3M Vetrap and elastic bands secured around the wing and silicone backpack worn for individual identification.

To partially immobilize hens, first, the tips of the flight feathers were tied together with two rubber bands (Staples Canada, Richmond Hill, ON, Canada). Second, 3M Vetrap (3M, Saint Paul, MN, USA) was wrapped around the flight feathers and rubber bands three times and then looped through the ‘backpack’ strap of that wing and wrapped a fourth time around the wing. Third, two more rubber bands were wrapped around the 3M Vetrap to secure it into place. Lastly, two rubber bands were used to wrap around the wing and the silicone ID backpack to keep the wing in place (figure 1). Slings were checked daily to ensure that worn or broken elastics were replaced and that the fit of each sling was not overly restrictive (e.g. approx. 1 cm of shoulder movement allowed). For full immobilization, birds (*n* = 20) were housed in an individual cage (length × width × height: 25.4 × 45.7 × 45.7 cm) with a feeder in front and water available through an overhead nipple drinker. Space required for wing flapping is greater than or equal to 1693 cm^2^ with a cage height of at least 49.5 cm [34,47], as such wing flapping was impaired in fully immobilized hens (1160.8 cm^2^/hen; 45.7 cm cage height).

### 2.3. Behavioural activity measurements

**Figure 2.**
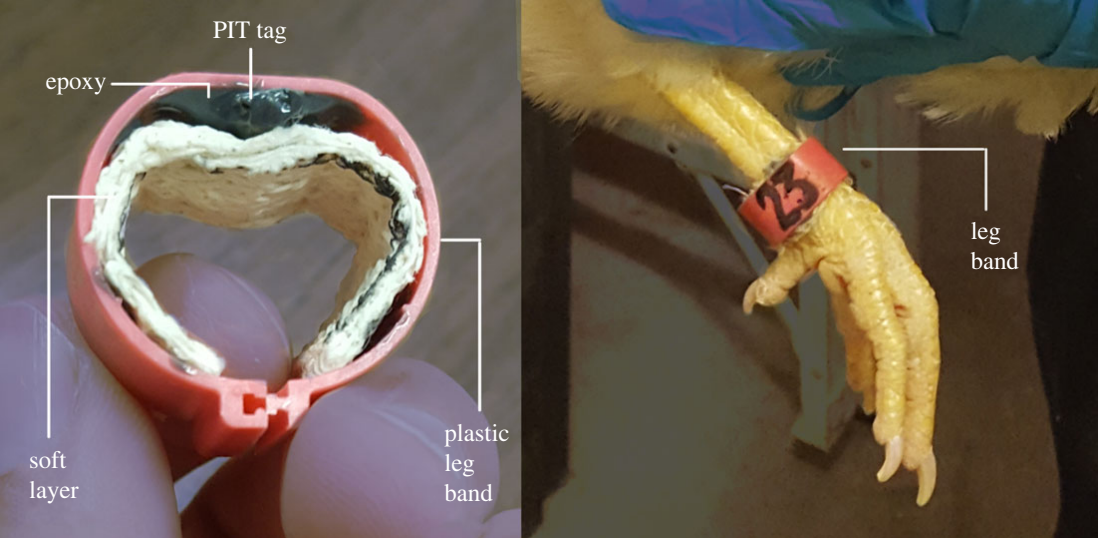
Plastic chicken leg bands (20 mm) modified to include a PIT tag encased within a layer of duct tape and bound to the band with a layer of steel-enforced epoxy. A soft layer of elastic bandage was included.

Behavioural activity measurements were only conducted in pens (i.e. non-immobilized or partially immobilized hens) as it was not possible in cages (i.e. fully immobilized hens). The amount of time that each hen spent at elevated and ground resources was measured with radio-frequency identification (RFID) technology (Biomark, Boise, ID, USA) before (week 0) and five weeks after treatment over a 48 h period. A passive integrated transponder (PIT) tag with a microchip containing a unique barcode was fixed to the plastic leg band (20 mm) of each hen (figure 2). Antennas producing a radio-frequency field were placed within individual nest-boxes and fixed to feeders. It was important to ensure that only one hen could access each resource at a time to prevent interference with RFID recordings. As described by Garant *et al.* [45], feeders were altered to include a duct-taped (Gorilla Tape; The Gorilla Glue Company, OH, USA) wrapped cardboard ring that limited the flow of feed to one position on each feeder. Following this, two wooden boards were attached to either side of this opening so that only one hen could enter the location where feed was available. This also ensured each hen would be required to step onto the antenna between the two wooden boards to access feed. When a hen would walk onto an antenna, the unique barcode of the PIT tag would be recognized by the antenna's radio-frequency field. These data would then be directly stored onto a microchip of the RFID unit as a time stamp (HH : MM : SS : MS) for every millisecond that a hen was accessing each resource.

### 2.4. Pectoralis, supracoracoideus and lower leg thickness

**Figure 3.**
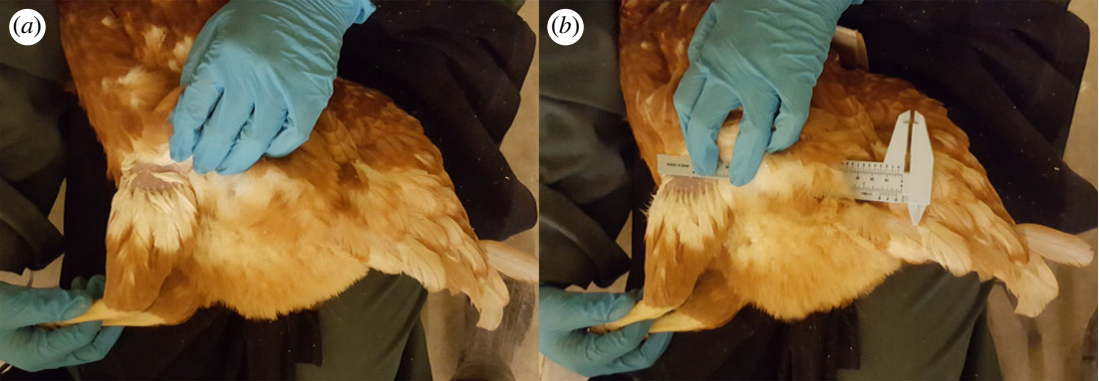
Placement of hens during ultrasound imaging of the lower leg. Hens were gently laid in the lap of a bird handler to image the left or right leg. The current figure shows placement for the left leg, the head of the hen is out of view at the top of the figure and the vent located in the right bottom of the figure. (*a*) The shank and lower leg were held such that they formed a 90° angle by holding the tarsometatarsus with one hand while the other hand gently parted the feathers. (*b*) A plastic vernier calliper was placed just below the knee and perpendicular to the length of the tibiotarsus and the transducer placed caudal to the calliper to obtain lower leg cross-section.

Directly before (week 0) and five weeks following immobilization treatment, cross-sectional images of the breast (pectoralis and supracoracoideus) and lower leg (*M. gastrocnemius pars medialis*, *M. fibularis lateralis* and *M. tibialis cranialis caput tibiale and femorale*) of each hen were obtained with a portable ultrasound unit (EBit 50 Unit, Digital Color Doppler Ultrasound System, Chison Medical Technologies, Bellevue, WA, USA). The procedure followed the same protocol as in Garant *et al.* [48]. In brief, each hen was securely held in the lap of an assistant while a trained second person captured ultrasound images. A multi-purpose ultrasound gel (Wavelength Ultrasound Gel (blue), National Therapy Products, Brampton, ON, Canada) was used as a contact agent and applied to the skin after gently parting the feathers. The assistant would position each hen on their back while restraining the feet so that the sternum was facing upwards to collect breast muscle images. Techniques used for transducer placement were adapted from Dietz *et al.* [49]. A plastic vernier calliper was used to ensure consistency with transducer placement. To image the flight muscles, the calliper was placed atop the left or right breast, perpendicular to the length of the keel bone and resting at the cranial apex of the keel. The calliper and keel bone made a 90° angle, and the transducer was placed such that it was caudal to the calliper and perpendicular to the keel bone. To image the lower legs (figure 3), hens were positioned laterally on their left (for left leg) or right side (for right leg) within the lap of the assistant. The feet were gently restrained, and the shank and lower leg were positioned to form a 90° angle. The calliper was placed perpendicular to the tibiotarsus, just below the knee and the transducer caudal to the calliper; this was repeated for both legs.

To measure the thickness of the muscles from the captured ultrasound images, the images were transferred from the ultrasound unit to a computer and analysed by a trained observer unaware of treatments with ImageJ software [50]. Thickness measurements were collected for the left and right muscles (pectoralis, supracoracoideus, lower leg muscles) of each bird. Thickness measurements were performed as described in Garant *et al.* [48]. In brief, the thickness of the pectoralis was measured between the skin and epimysial fascia, and the supracoracoideus between the epimysial fascia and keel bone. The thickness of the lower legs (cumulative measure of the *M. gastrocnemius pars medialis*, *M. fibularis lateralis* and *M. tibialis cranialis caput tibiale* *and femorale*) was measured between the skin of the lateral and medial sides of the lower leg.

### 2.5. Keel bone fracture and deviation in partially, fully and non-immobilized birds

The presence and severity of keel bone fractures were assessed from X-ray images as described in Garant *et al.* [48]. A Poskom VET-20BT portable X-ray unit (Promark Imaging, Toronto, ON, Canada) kept within a steel-doored, purpose concrete room (Research Station, University of Guelph) was used to obtain keel radiographs before (week 0), and five weeks post-treatment application. Imaging techniques were adapted from Woolcott *et al.* [51]. Image quality and density parameters of the X-ray unit were set to 90 kVp and 20 mAs, respectively. The receptor panel (image matrix of 19.65 × 23.6 cm, resolution of 77 µm @2560 × 3072 pixels) was placed within a fitted compartment of a custom-made wooden box and was approximately 80 cm from the X-ray unit. Additionally, the custom-made box contained evisceration shackles that were used to position and suspend hens in an upside-down position for approximately 10 s while obtaining images [52]. A built-in crank allowed for the vertical repositioning of the receptor panel to align the keel bone of each hen with the centre of the receptor panel. As images were taken, they were directly stored onto a computer connected to the X-ray unit via a 2.4 and 5.0 GHz wireless gigabit interface.

Following the acquisition, radiograph scoring was conducted by a trained, blinded observer for fracture severity and presence. Image scoring techniques, training and instruction were provided by Rufener *et al.* [53]. Keel bone fracture severity scores ranged from 0 (no fracture) to 5 (most severe). The presence of keel bone fractures was scored as a binary (0 = no fracture i.e. severity score 0; 1 = fracture present i.e. severity score greater than 0). The presence of keel bone deviations was determined with palpation. Hens were briefly suspended in an upside-down position. At the same time, a trained individual blinded to treatment used their thumb and forefinger to palpate the keel and feel for abnormality in its shape. Keel bone deviation presence was scored as a binary: 0—deviation absent or very mild, or 1—deviation present, detectable abnormality in keels structure [54].

### 2.6. Statistical analysis

All analyses were conducted in SAS v. 9.4 (SAS Institute Inc., Cary, NC, USA, 2012). Studentized residual plots were used to determine normally distributed residuals and homogeneity of variance. The Shapiro–Wilk statistic was used to determine the distribution of best fit. Degrees of freedom were calculated with a Kenward–Roger approximation, and multiple comparisons were accounted for with a Tukey–Kramer adjustment. Presented results (least squared (LS) means ± s.e., unless otherwise mentioned) with a *p*-value < 0.05 were considered statistically significant.

The behaviour observations were only possible for the partial and non-immobilized birds that remained in the floor pens. The proportion of time that each bird spent at the elevated resource (elevated feeder and elevated nest-box) out of the total time spent at the type of resource (feeder and nest-box) was calculated over 48 h. A generalized linear mixed model (PROC GLIMMIX) with a beta distribution was used to evaluate the effects of treatment (non-immobilized, partially immobilized), strain (white-feathered, brown-feathered), week (week 0, week 5 after treatment application) and all possible two-way and one three-way interaction on the proportion of time spent at the elevated feeder and nest-box. The model included pen as a random effect and accounted for repeated measures while considering hen within each pen as the experimental unit. Specific comparisons were used to firstly evaluate differences between strains at baseline (week 0) before treatments were applied. Secondly, as strain differences were found, subsequent analyses were sliced by strain and were focused to determine if there were differences in the percentage of time spent at each resource within the same strain and the same treatment, from week 0 to week 5.

Change in muscle thickness and body weight was calculated as the difference between muscle thickness at week 5 and baseline (week 0) expressed as a percentage of the baseline value at week 0 as per Garant *et al.* [48]. Thus, these percentages of change could be negative (indicating reduction) or positive (indicating increase). For partially immobilized birds, we analysed the ipsilateral and contralateral breast muscles separately, but only results from the ipsilateral muscles are presented (no changes observed in contralateral breast muscles). For the non- and fully immobilized birds, the left or right breast muscle was selected randomly with a random number generator, lower leg thickness values were an average measure of the left and right lower leg for all birds. Changes in muscle thickness of the pectoralis, supracoracoideus and lower leg muscles were analysed with a generalized linear mixed model (PROC GLIMMIX) using the fixed effects of treatment (no, partial and full wing immobilization), strain (white-feathered, brown-feathered) and treatment by strain interaction. This model was used to evaluate the percentage change in muscle thickness between the treatment groups, within each muscle group of each strain, and whether this percentage change of muscle thickness was significantly different from 0%. Bodyweight was analysed with the same model.

As only 10 out of 60 birds had a keel bone fracture score greater than 1 by week 5 (score 2: 11.70%, score 3: 5.00%, score 4: 0.00%, score 5: 0.00%), keel bone fracture severity was not further analysed. Instead, only the binary fracture presence outcome (0—no fracture, 1—fracture score of greater than 0) was analysed using a generalized linear mixed model (PROC GLIMMIX). The model calculated the odds of having a fracture with treatment (no, partial and full immobilization), strain (white-feathered, brown-feathered) and their interaction as the fixed effects while including the fracture status at week 0 as a covariate. However, due to the low number of birds with keel fractures within all categories of the interaction, specific contrasts were estimated to compare strains (white-feathered versus brown-feathered birds) and treatments (i.e. partially immobilized versus non-immobilized, fully immobilized versus non-immobilized). Results are presented as odds ratio (OR), and 95% confidence intervals (CIs), wherein a higher risk of keel bone fracture is indicated as OR greater than 1. As the number of hens with keel bone deviation was low and did not differ from week 0 to week 5 (*n* = 7), we did not further analyse this data.

## 3. Results

### 3.1. Behavioural activity before and after five weeks of no or partial immobilization using a wing sling

There was a significant difference in the amount of time that brown-feathered birds overall spent at the elevated feeder (12.50 ± 5.79%) compared with white-feathered birds (46.64 ± 7.35%) before treatment application (week 0) (*t*_21.03_ = −3.15, *p* = 0.0040). By contrast, the amount of time spent at the elevated nest-box did not differ between brown- and white-feathered birds before treatments were applied (week 0) (*t*_69_ = −0.75, *p* = 0.4595; 53.28 ± 14.47% versus 65.51 ± 15.55%).

Five weeks after birds were partially immobilized, there was no significant change, within strain, in the amount of time that brown- or white-feathered birds spent at the elevated feeder compared with week 0 (table 1). Both the non- and partially immobilized white-feathered hens saw a numerical decrease in time spent at the elevated feeder (−15.55% and −18.13%, respectively, from week 0). Due to this, the previously discussed initial difference in feeder usage observed between strains on week 0 was no longer present on week 5 (week 5 brown-feathered 14.41 ± 6.05% versus white-feathered 29.76 ± 6.74%; *t*_25.47_ = −1.67, *p* = 0.1067). Within strain, non-immobilized birds of both strains spent a similar amount of time at the elevated feeder and nest-box at weeks 0 and 5 (table 1). However, partial immobilization affected elevated nest-box usage in brown-feathered birds. We detected a 41.83% decrease in time that partially immobilized brown-feathered birds spent at the elevated nest-box on week 5 compared with week 0 (table 1; *t*_21.09_ = 2.86, *p* = 0.0346). While non-significant, partially immobilized white-feathered hens also saw a 28.14% decline in usage of the elevated nest-box from week 0 to 5. By contrast, the non-immobilized groups of the white- and brown-feathered birds showed non-significant increases in usage of the elevated nest-box from week 0 to 5 (increase of 8.84% and 5.26%, respectively; table 1).

**Table 1.** The percentage of time that white- and brown-feathered laying hens of two treatment groups (non- and partially immobilized) spent at an elevated resource (feeder, nest-box) over a 48 h period at baseline (week 0) and week 5. Within each treatment group, identical letters (A or B) represent the least squared means that are statistically similar to one another.

	white-feathered		brown-feathered	
	non-immobilized	partially immobilized	non-immobilized	partially immobilized
*percentage of time spent at elevated feeder (%)*				
week 0	47.5 ± 9.83^A^	45.8 ± 9.81^A^	9.3 ± 7.00^A^	16.5 ± 8.65^A^
week 5	32.0 ± 9.06^A^	27.6 ± 9.17^A^	14.1 ± 8.49^A^	14.7 ± 8.20^A^
*percentage of time spent at elevated nest-box (%)*				
week 0	61.5 ± 20.56^A^	69.4 ± 18.99^A^	52.6 ± 17.86^A^	54.0 ± 17.35^A^
week 5	70.3 ± 18.92^A^	41.2 ± 21.19^A^	57.8 ± 17.59^A^	12.2 ± 9.40^B^

### 3.2. Pectoral and leg muscle thickness and bodyweight in birds with no, partial and full wing immobilization

Descriptive measurements for muscle thickness measured by ultrasound and bodyweight are reported in table 2. Five weeks after white-feathered birds received the partial immobilization treatment, there were no significant changes to the thickness of the pectoralis (i.e. percentage change was not different from 0%; *t*_50.54_ = −0.50, *p* = 0.6207; table 3). By contrast, white-feathered birds that received the full immobilization treatment showed a significant pectoralis decrease of approximately 17.00% (*t*_30.0_ = −4.28, *p* = 0.0002; table 3). This thickness decrease differed from the partially immobilized birds (*t*_40.5_ = 2.67, *p* = 0.0274; table 3), but not the non-immobilized birds (*t*_40.5_ = −2.24, *p* = 0.0742; table 3). Within the brown-feathered strain, there were no significant changes to the thickness of the pectoralis for all three treatment groups, nor were there differences observed between immobilized and non-immobilized birds (table 3).

**Table 2.** The baseline (week 0) muscle thickness (mm) and bodyweight (g) of white- and brown-feathered laying hens before treatment application (non-, partially and fully immobilized). Muscle thickness, as measured with ultrasonography, includes the pectoralis, supracoracoideus and lower legs (cumulative measurement of *M. gastrocnemius pars medialis*, *M. fibularis lateralis* and *M. tibialis cranialis caput tibiale and femorale*). All variables are presented with the number of hens (*N*), mean, standard deviation (s.d.), minimum (min) and maximum (max) values.

variable	treatment	*N*	mean	s.d.	min	max
*white-feathered*						
pectoralis	non-immobilized	10	5.77	0.44	5.31	6.55
	partially immobilized	10	5.54	0.58	4.55	6.27
	fully immobilized	10	5.74	0.83	4.80	7.11
supracoracoideus	non-immobilized	10	11.42	0.53	10.55	12.44
	partially immobilized	10	11.51	0.47	10.90	12.44
	fully immobilized	10	11.21	0.77	9.89	12.19
lower leg	non-immobilized	10	22.73	1.06	21.11	24.09
	partially immobilized	10	21.94	1.15	19.72	23.42
	fully immobilized	10	21.36	1.97	17.74	24.79
bodyweight	non-immobilized	10	1723	99	1530	1856
	partially immobilized	10	1688	121	1448	1858
	fully immobilized	10	1695	136	1422	1948
*brown-feathered*						
pectoralis	non-immobilized	10	5.42	0.38	4.70	5.85
	partially immobilized	10	5.30	0.58	4.28	6.37
	fully immobilized	10	5.00	0.73	3.66	5.99
supracoracoideus	non-immobilized	10	11.51	0.58	10.45	12.15
	partially immobilized	10	11.61	0.73	10.45	12.62
	fully immobilized	10	11.19	0.69	10.14	12.35
lower leg	non-immobilized	10	26.59	1.79	24.19	30.21
	partially immobilized	10	26.99	0.91	25.99	28.81
	fully immobilized	10	26.50	1.12	25.05	28.47
bodyweight	non-immobilized	10	1951	127	1754	2134
	partially immobilized	10	2011	165	1790	2330
	fully immobilized	10	2064	162	1838	2366

**Table 3.** The percentage change in muscle thickness and bodyweight from baseline (week 0) to five weeks post-treatment application of white- and brown-feathered laying hens of three treatment groups (non-immobilized (non), partially immobilized (partial), fully immobilized (full)) presented as LS means ± s.e. Muscles presented include the pectoralis, supracoracoideus and lower leg (cumulative measurement of *M. gastrocnemius pars medialis*, *M. fibularis lateralis* and *M. tibialis cranialis caput tibiale and femorale*). Within each muscle group of each strain, LS means ± s.e. that share a letter superscript are not significantly different (*p* > 0.05) between treatments (*p* > 0.05) and italics values indicate a significant difference from baseline (week 0).

	white-feathered			brown-feathered		
	non	partial	full	non	partial	full
*change (%) from week 0 to 5*						
pectoralis	−4.40 ± 3.99^A,B^	−1.99 ± 3.99^A^	*−17.06* ± *3.99*^B^	−0.85 ± 3.99^A^	1.41 ± 3.99^A^	5.11 ± 3.99^A^
supracoracoideus	−1.69 ± 1.34^A^	*−4.33* ± *1.42*^A^	*−3.99* ± *1.34*^A^	−1.78 ± 1.34^A^	*−3.56* ± *1.34*^A^	*5.79* ± *1.42*^B^
lower leg	−1.03 ± 1.57^A^	−0.85 ± 1.57^A^	−2.86 ± 1.63^A^	0.50 ± 1.57^A^	−0.95 ± 1.57^A^	−2.75 ± 1.63^A^
bodyweight	2.72 ± 1.70^A^	−0.38 ± 1.70^A,B^	*−3.91* ± *1.72*^B^	3.20 ± 1.70^A^	0.46 ± 1.70^A^	3.07 ± 1.72^A^

More changes were observed in the supracoracoideus five weeks after treatment (table 3). Within the white-feathered strain, the partially and fully immobilized birds showed a significant decrease of approximately 4.00% to the thickness of the supracoracoideus (*t*_52.0_ = −3.06, *p* = 0.0035; and *t*_52.0_ = −2.97, *p* = 0.0045, respectively), though no differences between treatment groups were observed (all *p* > 0.05, table 3). By contrast, fully immobilized brown-feathered birds showed an increase in supracoracoideus thickness of nearly 6.00% (*t*_52.0_ = 4.09, *p* = 0.0002), while partially immobilized brown-feathered birds showed a decrease of nearly 4.00% (*t*_52.0_ = −2.56, *p* = 0.0106) and non-immobilized birds showed no change (*t*_52.0_ = −1.32, *p* = 0.1912). The increase observed in the fully immobilized brown-feathered birds differed significantly from the partially immobilized birds (*t*_52.0_ = −4.79, *p* < 0.0001) and non-immobilized birds of the brown-feathered strain (*t*_52.0_ = 3.88, *p* = 0.0009; table 3). No changes in lower leg muscle thickness were observed in white- and brown-feathered birds regardless of whether they were non-, partially or fully immobilized (table 3).

Average body weight of white-feathered birds was 1700 ± 110 while that of brown-feathered birds was 2100 ± 110 g (mean ± s.d.) at week 5. Within the white-feathered strain, there was a 4.00% decrease in bodyweight of fully immobilized birds (*t*_28.1_ = −2.27, *p* = 0.0313; table 3), while no change was observed in the non- and partially immobilized birds (table 3). As such, the reduction in bodyweight in fully immobilized birds was greater compared with the non-immobilized birds (*t*_38.3_ = −2.74, *p* = 0.0232; table 3), but not the partially immobilized birds (*t*_38.3_ = 1.46, *p* = 0.3207; table 3). Finally, for the brown-feathered strain, there were no significant changes to bodyweight five weeks after treatments were applied and no differences between treatments (table 3).

### 3.3. Keel bone fracture and deviation

At baseline (week 0), 14 hens had a keel bone fracture (23.30% flock prevalence; 11 white-feathered and three brown-feathered; table 4) that were mostly mild (92.90% with score 1 or 2). Five weeks after treatments were applied, 24 hens had a keel bone fracture (40.00% flock prevalence; table 4); 63.00% of these fractures were found in white-feathered hens, while 37.00% were observed in brown-feathered hens. Similar to week 0, the majority of fractures were still considered mild (87.50%). Birds were not more likely to have a keel bone fracture on week 5 compared with week 0 (OR = 2.5, 95% CI 0.91–5.98). There was no difference in the likelihood of having fractures between white- and brown-feathered birds five weeks after treatment application (OR = 1.95, 95% CI 0.51–7.44). Partially and fully immobilized birds were also equally as likely to have a keel bone fracture compared with the non-immobilized birds (partially immobilized: OR = 1.59, 95% CI 0.37–6.85; fully immobilized: OR = 0.26, 95% CI 0.05–1.50) following five weeks of treatment.

**Table 4.** The number of hens (*N*) with a keel bone fracture and keel bone deviation on weeks 0 and 5 within each strain (white- or brown-feathered birds, 30 birds per strain), treatment group (non-immobilized (non), partially immobilized (partial), fully immobilized (full), 20 birds per treatment) and strain by treatment combinations (10 birds per combination). Percentages are expressed out of the total number of birds within each respective group (strain, treatment, strain × treatment combination or total).

	keel fracture		keel deviation	
	week 0	week 5	week 0	week 5
strain				
white	11 (36.70%)	15 (50.00%)	3 (10.00%)	2 (6.70%)
brown	3 (10.00%)	9 (30.00%)	4 (13.30%)	5 (16.60%)
treatment				
non	5 (25.00%)	9 (45.00%)	3 (15.00%)	2 (10.00%)
partial	5 (25.00%)	11 (55.00%)	2 (10.00%)	4 (20.00%)
full	4 (20.00%)	4 (20.00%)	2 (10.00%)	1 (5.00%)
strain × treatment				
white—non	4 (40.00%)	7 (70.00%)	1 (10.00%)	1 (10.00%)
white—partial	4 (40.00%)	5 (50.00%)	1 (10.00%)	1 (10.00%)
white—full	3 (30.00%)	3 (30.00%)	1 (10.00%)	0 (0.00%)
brown—non	1 (10.00%)	2 (10.00%)	2 (20.00%)	1 (10.00%)
brown—partial	1 (10.00%)	6 (60.00%)	1 (10.00%)	3 (30.00%)
brown—full	1 (10.00%)	1 (10.00%)	1 (10.00%)	1 (10.00%)
total	14 (23.30%)	24 (40.00%)	7 (11.70%)	7 (11.70%)

The presence of deviations throughout the study did not change, with seven hens (11.70% flock prevalence) presenting with deviations at baseline (week 0) and five weeks after treatment application (table 4). Therefore, the odds of having a keel bone deviation were not further analysed.

## 4. Discussion

This study sought to investigate if a reduction in wing use through increasing degrees of wing immobilization would result in changes to resource usage, the thickness of the flight and leg muscles and induce changes to keel bone integrity (deviations and fractures) in two strains of laying hens (white- and brown-feathered).

Before treatment application, there was a pronounced difference in the level of elevated feeder usage between strains. Usage of this resource is a good indicator of aerial activity as hens will make multiple trips to eat throughout the day. Brown-feathered birds spent the majority of time eating from the ground feeder (greater than 85%) compared with white-feathered birds who used both feeders evenly. This may be related to physical differences between strains. White-feathered laying hens have a lighter body mass and smaller leg muscles (as also observed in this study) [40,41] and have a smaller wing-loading (mass of bird over wing area) burden compared with brown-feathered birds [45]. Brown-feathered hens also have been shown to have smaller flight muscles and less keel area compared with white-feathered hens [40]. Furthermore, brown-feathered chicks spent more time on the ground [42], and used aerial spaces and perches less than white-feathered chicks [43]. Physical limitations may result in decreased movement of brown-feathered birds to elevated areas when they have equal access to the same resources at the ground level. By contrast to feeders, nest-boxes are typically visited once or twice during egg-laying which is a highly motivated and time-dependent behaviour [55]. This may explain why brown-feathered birds still access elevated nest-boxes as also observed in our previous work [45]. The aforementioned physical differences may explain why immobilization appeared to affect the behaviour of brown-feathered birds more strongly than white-feathered birds. We detected a 41.83% decline in time that brown-feathered birds spent at the elevated nest-box after five weeks of partial immobilization. Previously, we found similar reductions in elevated nest-box usage after clipping of wing feathers as another method to limit wing use [45]. The partially immobilized brown-feathered birds probably shifted their behaviour to match their physical ability. The increase in ground nest-box usage by brown-feathered hens could have resulted in behavioural synchrony and aggregation of hens at this resource, a common phenomenon observed in laying hens [56–58]. However, this is unlikely, as the non-immobilized birds of both strains showed no change to their pattern of nest-box usage. Partially immobilized white-feathered birds also displayed a numerical decrease in time spent at the elevated resources, but the one-sided wing immobilization may not have been as effective for the more flight-equipped white-feathered birds. However, it is important to note that hens restricted to the usage of one wing were still able to access elevated resources (no treatment groups dropped down to 0% usage). This supports the importance of the hindlimbs, which are important in take-off [17,18] in aerial ascension.

We predicted that changes in behaviour would be associated with changes in muscle thickness. While five weeks of partial immobilization did not produce expected thickness reductions to the pectoralis nor thickness increases of the lower legs in both white- and brown-feathered birds, full wing immobilization (mimicking wing disuse) resulted in a reduction in the pectoralis, supracoracoideus and bodyweight of white-feathered laying hens. The relocation from a pen with abundant opportunity to exercise to a restrictive cage unit which impacted two locomotion systems was expected to more extremely influence muscles. As the space required for wing flapping is at least 1693 cm^2^ [34,47], fully immobilized birds, restricted to 1160.8 cm^2^/hen, would have been unable to flap their wings. This explains the observed reduction in flight muscles as it is known that the disuse of the wings leads to flight muscle atrophy in wild birds [22–25] and lower pectoralis weights in cage-reared hens [59]. In humans, severe immobilization in the form of bed rest results in the rapid deterioration of skeletal muscle mass within the legs and a less pronounced reduction in muscles of the upper limbs [11,12,15,60,61], because they are bipedal [62,63]. Surprisingly, lower leg muscle thickness was not affected by cage restriction in white-feathered hens in the present study, possibly because the space available was sufficient for standing [34,64], and the associated mechanical loading may have been sufficient to maintain leg muscle thickness in the absence of walking, running and jumping. By contrast to their white-feathered counterparts, brown-feathered caged birds only displayed a significant thickness increase of the supracoracoideus. Potentially, the ground-dwelling nature of brown-feathered birds [43,44,65] resulted in the minimal load-bearing activity of the wings prior to treatment, and the disuse of the wings did not differ enough to produce changes. Under partial immobilization, birds were still able to use one wing and walk around, which we hypothesized would reduce flight muscle (ipsilateral) but increase leg muscle thickness. Interestingly, both strains showed a significant reduction in the thickness of the supracoracoideus. The supracoracoideus functions primarily in the upstroke [16,19,20]. As the sling restricted this upwards motion, it is likely that the reduced mechanical load produced the observed outcome. However, as the sling prevented downwards movement as well, we expected similar thickness reductions in the pectoralis. Potentially, the sling allowed for small movements of the wing and shoulder, allowing some degree of flexion of the pectoralis that was sufficient to maintain thickness, but less so in the supracoracoideus, which was more elevated. Additionally, it may have been that the average position of the immobilized wing puts the supracoracoideus and pectoralis at different points along their length–tension curves. With the wing slightly elevated, the supracoracoideus may have been nearer its minimal functional length to produce contractile force [66]. Effects of immobilization can be counteracted by increased activity of the non-immobilized limbs, as shown in humans [67]. However, the thickness of the contralateral pectoralis (not presented) did not differ from that of the controls in our study, suggesting it was not exercised any more than in the non-immobilized birds. Similarly, Nightingale *et al.* [68] ruled out the presence of hypertrophy of non-immobilized wings in one-wing-splinted broiler chicks, as the humeral density of the non-immobilized wing did not differ from that of the control group. Additionally, no changes were observed in lower leg muscle thickness. This indicates that chickens are not likely to experience compensatory hypertrophy of the non-immobilized wing or leg muscles.

The skeleton of laying hens is weaker than that of wild birds due to daily egg-laying drawing more calcium from the bones than can be replenished [69]. As such, we anticipated that a reduction in wing usage would further weaken the bone due to a decrease in mechanical loading on the keel bone and result in more keel fractures and deviations in the case of unequal wing use through subsequently unequal force applied to the bone. However, the presence of keel bone fractures and deviations was not significantly different between non-, partially and fully immobilized birds after five weeks of treatment. As the pectoralis muscle did not change in thickness in response to partial immobilization, it is possible that there were no related changes to the integrity of the keel bone and no increased fracture risk. However, fully immobilized white-feathered hens showed an approximately 20% decrease in the thickness of the pectoralis but no increase in keel fractures. Birds in cage systems may show lower keel fracture rates, a finding often attributed to the inability of hens to fall and collide as purported in open housing systems; however, keel fractures are in fact present in all housing systems [70,71]. It is possible that the mechanism by which bone mass is increased (wing-loading) is also the mechanism driving the production of fractures. As such, by placing hens in cages where they were unable to bear substantial weight on their keel bones, a lack of mechanical strain on the keel resulted in no new fractures. It would have been interesting to investigate changes to the physical bone properties (e.g. bone breaking strength, bone ash content, bone mineral content, bone density) of the keel bone as a result of immobilization [68,72–74], but this was not possible without sacrificing birds. Keel deviations did not change over the experiment and were not common. It was expected that, in particular, the unequal wing use in partially immobilized birds would lead to unequally distributed force on the keel. While the number of deviations was highest in the partially mobilized group, the numbers were too low to analyse and make definite conclusions. The overall low number of birds with keel fractures (*n* = 24 overall, 40.00% at week 5) and deviations (*n* = 7 overall, 11.70% at week 5) in the study means that results should be interpreted with caution. The immobilization treatment employed in this study was in place for a period of five weeks and applied to hens that were 25 weeks old. Keel fractures have been shown to increase in number and severity with age [36,75,76], corresponding to a decrease in bone integrity as cortical bone depletes [37]. Applying the treatment on older hens or leaving the treatment in place for a longer period of time would further elucidate the effects of wing disuse on keel bone fractures or deviations.

## 5. Conclusion

Altogether, white- and brown-feathered strains of laying hens are different in their behavioural and physical adaptations to wing immobilization. Brown-feathered birds showed a preference for the ground feeder and responded to partial immobilization with a significant decrease in elevated nest-box usage, while white-feathered birds did not. Furthermore, full immobilization had the opposite effects in the two strains: white-feathered hens saw reductions in pectoralis thickness and bodyweight, while brown-feathered hens showed increases. However, the presence of keel bone fractures and deviations was similar for both strains and not affected by the immobilization treatments.

## Data Availability

Raw data have been made publicly available as electronic supplementary material [77].
